# Genetic variation and forensic efficiency of 30 indels for three ethnic groups in Guangxi: relationships with other populations

**DOI:** 10.7717/peerj.6861

**Published:** 2019-05-03

**Authors:** Weian Du, Chunlei Feng, Ting Yao, Cheng Xiao, Hongyan Huang, Weibin Wu, Linnan Zhu, Honghua Qiao, Chao Liu, Ling Chen

**Affiliations:** 1 School of Forensic Medicine, Southern Medical University, Guangzhou, China; 2 Guangdong Homy Genetics Incorporation, Foshan, China; 3 Guangzhou Forensic Science Institute, Guangzhou, China

**Keywords:** DIPplex, Zhuang, Kelao, Yao, Guangxi, Insertion/deletion, Forensic genetics

## Abstract

**Aim:**

In this study, we used a series of diallelic genetic marker insertion/deletion polymorphism (indel) to investigate three populations of Yao, Kelao, and Zhuang groups in the Guangxi region of China and to evaluate their efficiency in forensic application.

**Result:**

No deviations for all 30 loci were observed from the Hardy–Weinberg equilibrium after Bonferroni correction (*p* > 0.05/30 = 0.0017). The allele frequencies of the short allele (DIP-) for the above three populations were in the range of 0.0520–0.9480, 0.0950–0.8780, and 0.0850–0.915, respectively. The observed heterozygosity of the 30 loci for the three populations was in the ranges 0.0802–0.5802, 0.1908–0.6053, and 0.1400–0.5600, respectively. The cumulative power of exclusion and combined discrimination power for Yao, Kelao, and Zhuang groups were (0.9843 and 0.9999999999433), (0.9972 and 0.9999999999184), and (0.9845 and 0.9999999999608), respectively. The DA distance, principal component analysis, and cluster analysis indicated a clear regional distribution. In addition, Zhuang groups had close genetic relationships with the Yao and Kelao populations in the Guangxi region.

**Conclusion:**

This study indicated that the 30 loci were qualified for personal identification; moreover, they could be used as complementary genetic markers for paternity testing in forensic cases for the studied populations.

## Introduction

Autosomal short tandem repeat (STR) is a standard marker for DNA analysis in forensic practice. However, STR mutations have always troubled the paternity testing. Compared to the mutation rates of STR (about 10^–3^), Insertion/deletion polymorphism (indel) markers have significantly lower mutation rates (less than 10^–8^) (Sobrino & Carracedo, 2005). However, single nucleotide polymorphisms (SNPs) have a low mutation rate, it is based on the sequence polymorphism rather than length polymorphism that is difficult to achieve simple and rapid genotyping in the routine forensic lab (Weber et al., 2002). Insertion/deletion polymorphisms are prospective genetic markers and spread widely throughout the genome (Nachman & Crowell, 2000); moreover, they also combine the desirable characteristics of both STRs and SNPs. Insertion/deletion polymorphism is a length polymorphism marker and it can be easily analyzed with capillary electrophoresis (Pereira et al., 2009). In totality, indels are considered to be promising markers in forensic application.

The Investigator DIPplex kit was the first commercial kit developed by Qiagen (Qiagen, Hilden, Germany) for the indel marker. This kit contains 30 autosomal indels and amelogenin, and it has been used to perform systematic investigations on worldwide populations of African (Hefke, Davison & D’Amato, 2015), Asian (Wang et al., 2014; Li et al., 2013; Liang et al., 2013; Meng et al., 2015; Seong et al., 2014; Wei et al., 2014; Yang et al., 2017), European (Friis et al., 2012; Kis et al., 2012; Martin et al., 2013), and American (Saiz et al., 2014) origins. However, very few studies have reported about how indels can be used in comprehending the genetic variation of ethnic minority groups (Zhuang, Yao, and Kelao) in the Guangxi Zhuang Autonomous Region, China. Therefore, we used the kit to understand population polymorphism of Yao, Kelao, and Zhuang ethnic groups in the Guangxi region and to evaluate the efficacy of this kit. Moreover, the genetic differentiation in the various populations was analyzed by comparing the results of the studied populations with those of other reported populations.

Guangxi Zhuang Autonomous Region is located in southwest China and adjacent to Guangdong, Guizhou, Yunnan, and Hunan provinces. One of the settlements of Chinese ethnic minorities is located in this region; moreover, the immigration and symbiosis of multiple ethnic groups have witnessed a fine balance in this region. Among the 11 ethnic minorities in the Guangxi region, the Zhuang group is the native inhabitant of Guangxi region; the tribe members are descendants of the Xi’ou and Luo’Yue clans that have been distributed in Guangxi since ancient times (Sun et al., 2013). The Zhuang is the largest ethnic minority group in China, about 87.77% of the Zhuang population is concentrated in the Guangxi region. Furthermore, the Kelao group migrated to the Guangxi region from Guizhou province, and the exodus of this tribe happened hundreds of years ago as this population group was fleeing from the famine-affected region of Guizhou in those times (Sun et al., 2013). During the reign of the erstwhile Sui and Tang dynasties, the Yao group moved to Guangxi from the present-day provinces of Hunan and Guangdong in China (Sun et al., 2013). According to the data released in 2010 census, the populations of the ethnic groups, Zhuang, Yao, and Kelao, are about 14.82 million, 1.349 million, and 2,900 people in the Guangxi region, respectively; the Yao is second only to the Zhuang group in terms of population sizes, while the population of the Kelao group is the lowest among the 11 ethnic groups of Guangxi region. (http://www.stats.gov.cn/english/statisticaldata/censusdata/rkpc2010/indexch.htm). Moreover, the Zhuang language, Yao language, and Kelao language all belong to the Sino-Tibetan language. In this study, the genetic data of these myriad types of populations based on 30 indels was investigated, and the results were further analyzed to evaluate whether they could be used in forensic applications and population genetic studies.

## Methods and materials

### Sample collections and DNA extraction

Bloodstain samples were collected from unrelated volunteers living in the Guangxi Zhuang Autonomous Region of China. The population sample sizes were as follows: Yao (*N* = 162), Kelao (*N* = 152), and Zhuang (*N* = 200). As described earlier by Walsh, Metzger & Higushi (2013), the Chelex-100 method was used for the extraction of genomic DNA from bloodstain samples. Informed consent was obtained from all the individuals and this study was approved by School of Forensic Medicine, Southern Medical University.

### Genotyping and amplification

In this study, PCR amplification was performed using the Investigator DIPplex kit handbook in accordance with the manufacturer’s instructions (http://www.qiagen.com/products/investigatordipplexkit.aspx) in a GeneAmp PCR system 9700 thermal cycler (Applied Biosystems, Foster City, CA, USA). The PCR products were detected with ABI 3130XL Genetic Analyzer (Applied Biosystems, Foster City, CA, USA). Genotypes were determined by GeneMapper ID-X software v1.3 (Applied Biosystems, Foster City, CA, USA). In addition, 9948 DNA (Promega, Fitchburg, WI, USA) was employed as a positive control in amplification.

### Quality control

We performed the study in accordance with the ISFG recommendations for DNA polymorphism analysis, and the procedure was described by Schneider (2007).

### Statistical analyses

Allele frequencies, Hardy–Weinberg equilibrium (HWE), observed heterozygosity (Ho), power of exclusion (PE), discrimination power (DP), match probability (MP), polymorphic information content (PIC), typical paternity index (TPI) were calculated using the modified powerstate (version 1.2) spreadsheet. Single nucleotide polymorphism Analyzer v2.0 was used to analyze linkage disequilibrium (LD) (Yoo et al., 2008). DISPAN program was used to calculate DA distances (Kim & Sappington, 2013). Principal component analysis (PCA) was based on allele frequencies, and it was carried out in MATLAB 2007a (MathWorks Inc., Natick, MA, USA). In addition, the STRUCTURE program (version 2.2) was used to analyze the population structure.

## Results

### Forensic parameters

The allele frequencies and forensic parameters of 30 indel loci in the three ethnic populations are presented in Table S1. The allele frequencies of the short allele for Yao, Kelao, and Zhuang ethnicities were in the range of 0.0520–0.9480, 0.0950–0.8780, and 0.0850–0.9150, respectively. The PE for the 30 indels was less than 0.3 with respect to Yao, Kelao, and Zhuang groups; thus, the PE value was much lower than those of most STRs (Deng et al., 2007; Guo, 2017; Jin et al., 2004). This implies that more loci are needed in indels to achieve the same value of the cumulative power of exclusion (CPE) than that for STR. The highest DP was observed at the following positions for the three groups: HLD48 (DP = 0.6520) for Yao, HLD48 (DP = 0.6111) for Kelao, and HLD56 (DP = 0.6494) for Zhuang. The polymorphism information content (PIC) was in the range of 0.0945–0.3750, and 67% of PIC values (20 out of 30) was over 0.3 in Yao samples. Then, the PIC values were in the range of 0.1577–0.3750, and more than 77% (23 out of 30) was over 0.3 in Kelao samples. Finally, the PIC values were in the range of 0.1435–0.3750, and more than 70% (21 out of 30) was over 0.3 in Zhuang samples. The above data indicate that polymorphism of indels is lower than STR; this occurs because an indel is a diallele with only two alleles per locus. The Ho for Yao, Kelao, and Zhuang samples are in the ranges 0.0802–0.5802, 0.1908–0.6053, and 0.1400–0.5600, respectively. The Ho of the seven loci in Yao samples, six loci in Kelao samples, and eight loci in Zhuang samples were less than 0.3 per experimental observations. The results indicate that some indel markers are relatively polymorphic while some indels exhibit rather low levels of polymorphism in the studied populations, such as HLD39, HLD64, HLD99, HLD111, and HLD118.

In the three populations, the departures from the HWE were observed at the HLD125 locus in Yao population, whereas the same parameter was observed at HLD45, HLD83, HLD114, HLD124, and HLD136 loci in Kelao population. After applying the Bonferroni correction (*p* > 0.05/30 = 0.0017), we could not observe any deviation from HWE. As displayed in Table S2, the CPE and CDP for the Yao, Kelao, and Zhuang samples were (0.9843 and 0.9999999999433), (0.9972 and 0.9999999999184), and (0.9845 and 0.9999999999608), respectively. A high level of DP was observed in the three sample populations (CDP > 0.9999), indicating the sufficient potential of 30 indels in forensic individual identification. However, the CPE was less than 0.9999, which indicates that 30 indels were not effective enough for paternity testing. Therefore, the DIPplex kit can serve as a supplement to the current STR system for paternity testing.

### Linkage disequilibrium analysis

**Figure 1:**
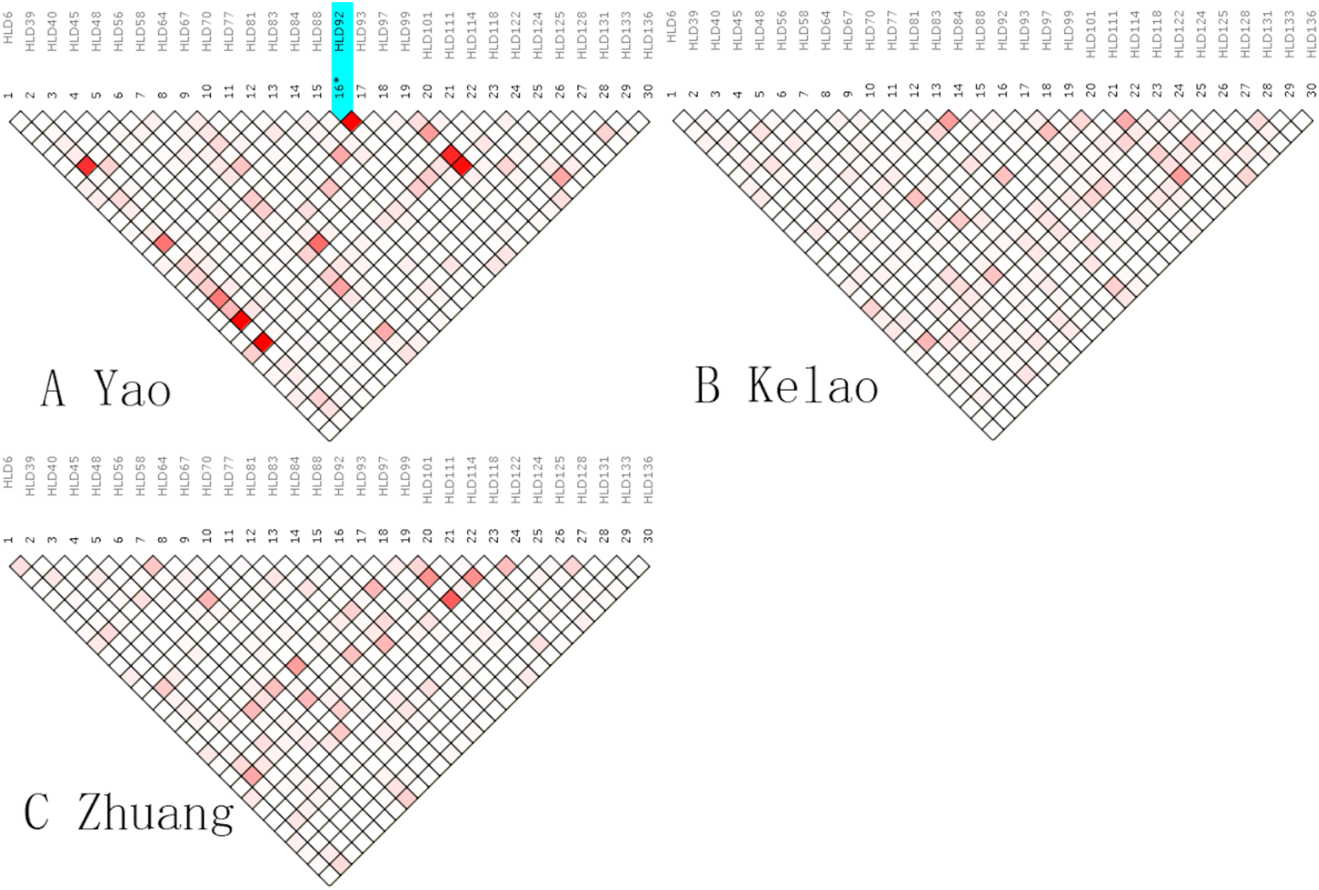
The linkage disequilibrium pattern of (A) Yao, (B) Kelao and (C) Zhuang.

An analysis of linkage disequilibrium that occurs between 30 indels was performed by SNPAnalyzer software. As is shown in Fig. 1, the pattern of linkage disequilibrium was revealed in a reverse triangle, and the intensity of red color in the plot is a measure of whether the linkage disequilibrium is strong in magnitude. The level of LD between 30 indel loci was estimated using *r*^2^ tested by the SNPs Analyzer program. With only HLD88 and HLD92 (*r*^2^ = 1) as exceptions, the *r*^2^ values of all loci were less than 0.8 in Yao samples. This means that HLD88 were linked with HLD92 in the Guangxi Yao samples. This probably occurs because the sample size of Yao individuals is too small.

### Population comparisons

**Figure 2:**
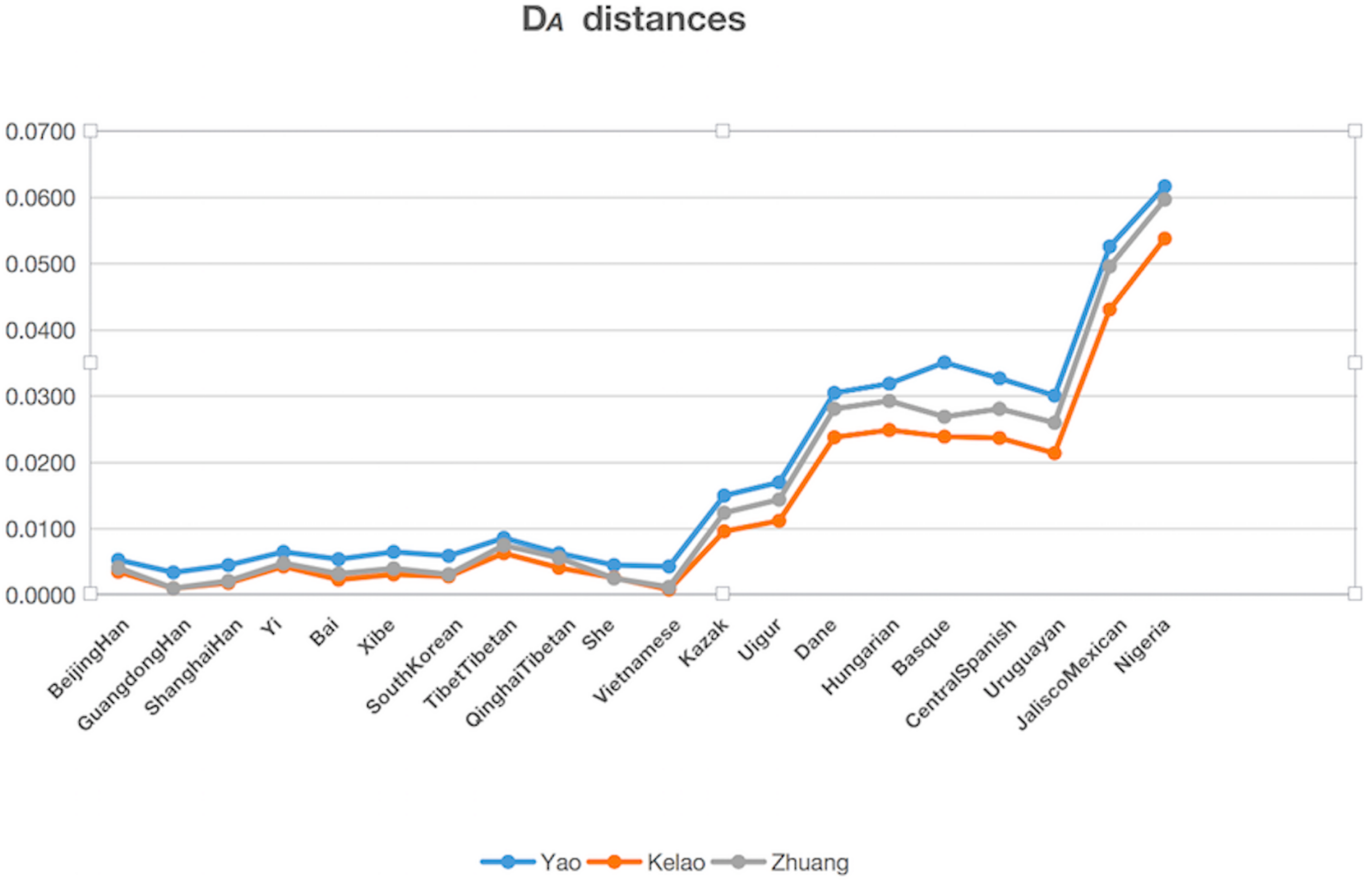
The DA distances among the Yao, Kelao and Zhuang groups and other 20 reference groups.

The genetic distances (D_*A*_ distance) were calculated among Yao, Kelao, and Zhuang samples, and the 20 referenced groups included Beijing Han (Wei et al., 2014), Guangdong Han ( Li et al., 2013), Shanghai Han (Wang et al., 2014), Yi (Liang et al., 2013), Bai (Yang et al., 2017), Xibe (Meng et al., 2015), South Korean (Seong et al., 2014), Tibet Tibetan (Wei et al., 2014), Qinghai Tibetan (Wei et al., 2014), She (Martin et al., 2013), Kazak (Wei et al., 2014), Uigur (Wei et al., 2014), Danes (Friis et al., 2012), Hungarian (Kis et al., 2012), Basque (Martin et al., 2013), Central Spanish (Martin et al., 2013), Uruguaya (Saiz et al., 2014), Jalisco Mexican (Martinez-Cortes et al., 2016), Nigeria (Du et al., 2017), Vietnamese (Du et al., 2017). The result of D_*A*_ distances was illustrated in Fig. 2, and it showed the Yao, Kelao, and Zhuang groups have a close genetic distance with most Asian populations, especially the Guangdong Han and Vietnamese populations. Guangdong and Vietnam are adjacent to Guangxi which enables easy migration into Guangxi for work or marriage. Thus, geographical factors may help explain why the Guangxi three ethnic groups have close genetic relations with Guangdong Han and Vietnamese populations.

### Principal component analysis

**Figure 3:**
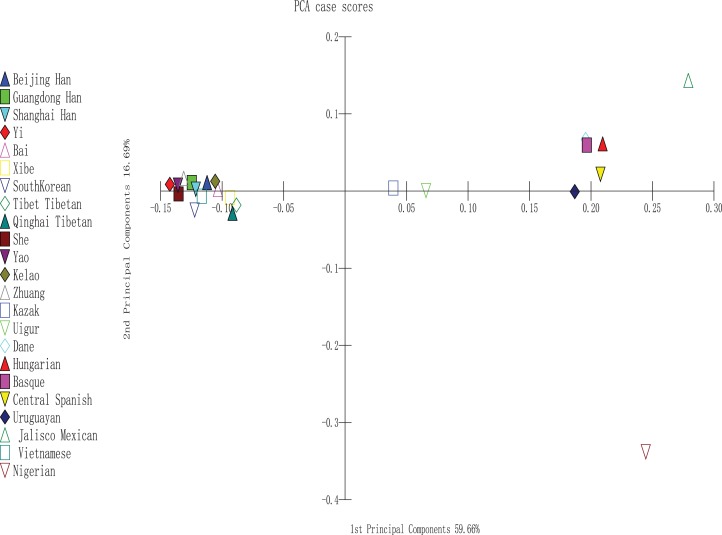
PCA based on 30 indel loci of the Yao, Kelao and Zhuang groups and other 20 reference groups.

To perform PCA, we used MATLAB 2007a software (Tan et al., 2014); the analysis was completely based on the allele frequencies of Yao, Kelao, Zhuang, and 20 referenced groups. The proportions of first and second components were 59.66% and 16.69%, respectively. In addition, the two components occupied 76.35% of the total variance. As shown in Fig. 3, the result indicated a clear regional distribution. In addition, Yao, Kelao, Zhuang, and the other 11 East Asian groups were located on the left midline of the distribution, whereas Kazak and Uighur were located on the right middle section. Four European groups were located on the upper right quadrant near the midline and close to the Uruguayan group, whereas Jalisco Mexican group was located on the right upper quadrant. In addition, Nigeria was located on the lower right quadrant.

### Population STRUCTURE analysis

**Figure 4:**
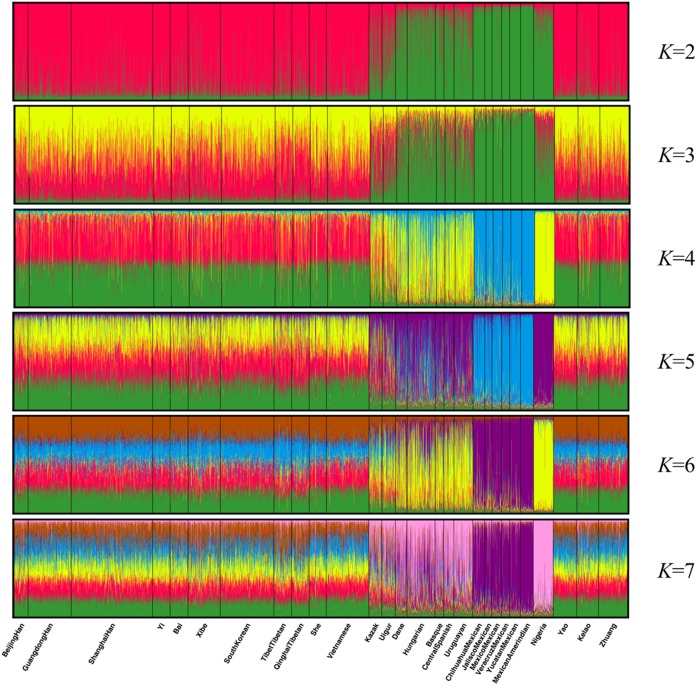
Clustering analysis by STRUCTURE for the full-loci dataset assuming *K* = 2–7.

Population structure analysis was performed using the STRUCTURE program with 10,000 burn-in period and additional 10,000 Markov Chain Monte Carlo replication; and the *K* values were set to 2–7 and each *K* was run in 15 replicates (Falush, Stephens & Pritchard, 2003). As shown in Fig. 4, the 28 groups were clarified clearly into distinct geographic patterns. When *K* at 2 and 3, East Asian groups, North American groups and European groups could be differentiated by distinct discrepancy of color compositions. Statistically, *K* = 4 is the best *K* value. When *K* at 4, the constituents of three populations of Guangxi region and the other 11 East Asian groups exhibited a mixture of red and green, whereas the six components of North American group are almost entirely blue. Meanwhile, the four European groups and Uruguayan group exhibited a mixture of yellow, blue, and green components. Meanwhile, Eurasia populations (Kazak and Uigur groups) also exhibited a mixture of yellow, blue, and green components, where in the proportion of green component was higher. In addition, the main constituent is yellow in the African groups. At *K* > 4, we did not observe any further substructure. Hence, the 30 indels can distinguish ancestries of the studied population and other populations to some extent; however, this result warrants further investigation.

## Discussion

### Alleic polymorphisms and forensic statistical parameter analysis

The allele frequencies of 30 indel loci could distinguish different populations to a certain degree, and the results revealed that Guangxi three ethnic groups were similar to the East Asians, especially with most Chinese populations in allele frequency distributions. In the forensic application field, CPE and CPD values are common indicators to estimate the forensic efficiency. In this study, the CPE and CPD values for the Yao, Kelao, and Zhuang samples were 0.9843 and 0.9999999999433, 0.9972 and 0.9999999999184, and 0.9845 and 0.9999999999608, respectively. The high CPD value clarified that these 30 indel loci could be regarded as efficient genetic markers in forensic identification cases, while the relatively lower CPE value indicated that the 30 loci could only be used as complementary genetic markers for paternity testing.

### Linkage disequilibrium analysis

The indel loci are suitable for forensic applications as independent loci if no relevance exists between two loci from the same chromosome or two random chromosomes (Slatkin, 2008). The LD analysis indicated no significant LD among the 30 loci in Zhuang and Kelao groups, while HLD88 were linked with HLD92 in Yao sample. This probably occurs because the sample size of Yao individuals is too small. Beyond that, there was no significant LD among the loci which indicated independence from the other 30 indels in the Guangxi Zhuang and Kelao groups. Nonetheless, it is worth noting that the sample size is limited, and further studies are needed to help us to confirm our findings.

### Population differentiations

Genetic distance reflected by D_*A*_ distance is considered an effective analysis method to reveal the genetic divergences between populations within a species (Nei, Tajima & Tateno, 1983). Detailed data were shown in Dataset S4, and the genetic distance between Yao and Zhuang, Yao and Kelao, and Zhuang and Kelao were 0.0042, 0.0042, and 0.0011, respectively; thus, the genetic distances between Yao, Kelao, and Zhuang groups were very close. The results show the Kelao groups had closer relationships with Zhuang groups, indicating that Kelao groups in the Guangxi region might have been subjected to intensive gene exchange with Zhuang groups in ancient times (He et al., 2019). As shown in Fig. 2, the three Guangxi groups have a close genetic distance with most Asian populations, the values of *D*_*A*_ genetic distance were consistent with the geographic locations of these populations. Compared with East Asian groups, Eurasian groups, European groups, Mexican groups, and Nigeria groups were found had more differences with Guangxi three ethnic groups. The results of the STRUCTURE analysis were roughly in line with population differentiation. From this analysis, 28 populations were classified into geographic patterns.

### Principal component analysis

According to Fig. 3, different continent populations could be divided into four quadrants. Most Asian groups (Beijing Han, Shanghai Han, Guangdong Han, Xibe, Yi, Bai, Zhuang, Yao, Kelao, She, Tibet Tibetan, Qinghai Tibetan, South Korean, and Vietnamese groups) distributed in the left quadrant. The studied Yao and Zhuang groups were adjacent to She, Yi, and Guangdong Han groups, and Kelao group was adjacent to Bai groups. Following by other Chinese populations, which indicated Guangxi three ethnic groups might have close genetic relationships with She, Yi, Guangdong Han, and Bai groups. In addition, Yao groups had closer relationships with She groups. It may be that Yao groups and She groups have a homologous ancestor (Li et al., 2003). Moreover, the distribution of the other populations was in accordance with geographical patterns.

## Conclusion

In conclusion, we obtained the allele frequencies and forensic parameters of 30 autosomal indels loci for Yao, Kelao, and Zhuang groups in Guangxi. The set of 30 indels showed high efficiency in individual identification of these samples. The results of D_*A*_ distance, PCA, and cluster analysis all showed that Zhuang groups had very close genetic relationships with Yao and Kelao groups in the Guangxi region. Furthermore, studies were performed in order to compare the three ethnic groups in Guangxi with more reference groups. All these results were helpful in providing a better understanding of the genetic background of people in the Guangxi region.

## Supplemental Information

10.7717/peerj.6861/supp-1 Supplemental Information 1 Raw data of the 30 indel loci in Zhuang group.

10.7717/peerj.6861/supp-2 Supplemental Information 2 Raw data of the 30 indel loci in Yao group.

10.7717/peerj.6861/supp-3 Supplemental Information 3 Raw data of the 30 indel loci in Kelao group.

10.7717/peerj.6861/supp-4 Supplemental Information 4 The dataset of DA distance.

10.7717/peerj.6861/supp-5 Supplemental Information 5 Allele frequencies and forensic parameters of the 30 INDELs in Guangxi three ethnic individuals.

10.7717/peerj.6861/supp-6 Supplemental Information 6 Parameters of genetic diversity and forensic efficiency for the 30 INDELs in Guangxi three ethnic individuals.

## Additional Information and Declarations

### Data Availability

The following information was supplied regarding data availability: The raw measurements and raw data are available in the Supplemental Files.

## References

[ref-24] Deng Q, Xu L, Gong J, Zhou L, Li S, Deng X, Luo G, Xie X (2007). Genetic relationships among four minorities in Guangxi revealed by analysis of 15 STRs. Journal of Genetics and Genomics.

[ref-28] Du W, Peng Z, Feng C, Zhu B, Wang B, Wang Y, Chao L, Chen L (2017). Forensic efficiency and genetic variation of 30 InDels in Vietnamese and Nigerian populations. Oncotarget.

[ref-30] Falush D, Stephens M, Pritchard JK (2003). Inference of population structure using multilocus genotype data: linked loci and correlated allele frequencies. Genetics.

[ref-13] Friis SL, Borsting C, Rockenbauer E, Poulsen L, Fredslund SF, Tomas C, Morling N (2012). Typing of 30 insertion/deletions in Danes using the first commercial indel kit—Mentype® DIPplex. Forensic Science International: Genetics.

[ref-25] Guo F (2017). Genetic variation of 17 autosomal STR loci in the Dong ethnic minority from Guangxi Zhuang Autonomous Region, South China. International Journal of Legal Medicine.

[ref-5] Hefke G, Davison S, D’Amato ME (2015). Forensic performance of Investigator DIPplex indels genotyping kit in native, immigrant, and admixed populations in South Africa. Electrophoresis.

[ref-26] Jin TB, Gao Y, Yan CX, Chen T, Li SB (2004). Genetic relationship between minorities in Guangxi by STR markers. Zhong Nan Da Xue Xue Bao Yi Xue Ban.

[ref-33] He G, Wang Z, Zou X, Wang M, Liu J, Wang S, Ye Z, Chen P, Hou Y (2019). Tai-Kadai-speaking Gelao population: Forensic features, genetic diversity and population structure. Forensic Sci Int Genet.

[ref-22] Kim KS, Sappington TW (2013). Microsatellite data analysis for population genetics. Methods in Molecular Biology.

[ref-14] Kis Z, Zalan A, Volgyi A, Kozma Z, Domjan L, Pamjav H (2012). Genome deletion and insertion polymorphisms (DIPs) in the Hungarian population. Forensic Science International: Genetics.

[ref-34] Li H, Pan W, Wen B, Yang N, Jin J, Jin L, Lu D (2003). Origin of Hakka and Hakkanese: a genetics analysis. Acta Genetica Sinica.

[ref-7] Li H, Xiao-guang W, Su-juan L, Yin-ming Z, Xue-ling O, Yong C, Wei-hong C, Hong-yu S (2013). Genetic polymorphisms of 30 Indel loci in Guangdong Han population. Journal of Sun Yat-sen University(Medical Sciences).

[ref-8] Liang W, Zaumsegel D, Rothschild MA, Lv M, Zhang L, Li J, Liu F, Xiang J, Schneider PM (2013). Genetic data for 30 insertion/deletion polymorphisms in six Chinese populations with Qiagen Investigator DIPplex Kit. Forensic Science International: Genetics Supplement Series.

[ref-15] Martin P, Garcia O, Heinrichs B, Yurrebaso I, Aguirre A, Alonso A (2013). Population genetic data of 30 autosomal indels in Central Spain and the Basque Country populations. Forensic Science International: Genetics.

[ref-27] Martinez-Cortes G, Garcia-Aceves M, Favela-Mendoza AF, Munoz-Valle JF, Velarde-Felix JS, Rangel-Villalobos H (2016). Forensic parameters of the Investigator DIPplex kit (Qiagen) in six Mexican populations. International Journal of Legal Medicine.

[ref-9] Meng H-T, Zhang Y-D, Shen C-M, Yuan G-L, Yang C-H, Jin R, Yan J-W, Wang H-D, Liu W-J, Jing H, Zhu B-F (2015). Genetic polymorphism analyses of 30 InDels in Chinese Xibe ethnic group and its population genetic differentiations with other groups. Scientific Reports.

[ref-3] Nachman MW, Crowell SL (2000). Estimate of the mutation rate per nucleotide in humans. Genetics.

[ref-32] Nei M, Tajima F, Tateno Y (1983). Accuracy of estimated phylogenetic trees from molecular data. II. Gene frequency data. Journal of Molecular Evolution.

[ref-4] Pereira R, Phillips C, Alves C, Amorim A, Carracedo Á, Gusmão L (2009). Insertion/deletion polymorphisms: a multiplex assay and forensic applications. Forensic Science International: Genetics Supplement Series.

[ref-16] Saiz M, André F, Pisano N, Sandberg N, Bertoni B, Pagano S (2014). Allelic frequencies and statistical data from 30 INDEL loci in Uruguayan population. Forensic Science International: Genetics.

[ref-20] Schneider PM (2007). Scientific standards for studies in forensic genetics. Forensic Science International.

[ref-10] Seong KM, Park JH, Hyun YS, Kang PW, Choi DH, Han MS, Park KW, Chung KW (2014). Population genetics of insertion–deletion polymorphisms in South Koreans using Investigator DIPplex kit. Forensic Science International: Genetics.

[ref-31] Slatkin M (2008). Linkage disequilibrium—understanding the evolutionary past and mapping the medical future. Nature Reviews Genetics.

[ref-1] Sobrino B, Carracedo A (2005). SNP typing in forensic genetics: a review. Methods in Molecular Biology.

[ref-17] Sun H, Zhou C, Huang X, Liu S, Lin K, Yu L, Huang K, Chu J, Yang Z (2013). Correlation between the linguistic affinity and genetic diversity of Chinese ethnic groups. Journal of Human Genetics.

[ref-29] Tan CS, Ting WS, Mohamad MS, Chan WH, Deris S, Shah ZA (2014). A review of feature extraction software for microarray gene expression data. BioMed Research International.

[ref-19] Walsh PS, Metzger DA, Higuchi R (2013). Chelex 100 as a medium for simple extraction of DNA for PCR-based typing from forensic material. BioTechniques.

[ref-6] Wang Z, Zhang S, Zhao S, Hu Z, Sun K, Li C (2014). Population genetics of 30 insertion–deletion polymorphisms in two Chinese populations using Qiagen Investigator(R) DIPplex kit. Forensic Science International: Genetics.

[ref-2] Weber JL, David D, Heil J, Fan Y, Zhao C, Marth G (2002). Human diallelic insertion/deletion polymorphisms. American Journal of Human Genetics.

[ref-11] Wei Y-L, Qin C-J, Dong H, Jia J, Li C-X (2014). A validation study of a multiplex INDEL assay for forensic use in four Chinese populations. Forensic Science International: Genetics.

[ref-12] Yang C-H, Yin C-Y, Shen C-M, Guo Y-X, Dong Q, Yan J-W, Wang H-D, Zhang Y-D, Meng H-T, Jin R, Chen F, Zhu B-F (2017). Genetic variation and forensic efficiency of autosomal insertion/deletion polymorphisms in Chinese Bai ethnic group: phylogenetic analysis to other populations. Oncotarget.

[ref-21] Yoo J, Lee Y, Kim Y, Rha S, Kim Y (2008). SNPAnalyzer 2.0: a web-based integrated workbench for linkage disequilibrium analysis and association analysis. BMC Bioinformatics.

